# Do the trade credit influence firm performance in agro-industry? Evidence from Thailand

**DOI:** 10.1016/j.heliyon.2023.e14561

**Published:** 2023-03-14

**Authors:** Umawadee Detthamrong, Wirapong Chansanam

**Affiliations:** a Department of Business and Innovation Management, Faculty of Business Administration, Chaiyaphum Rajabhat University, Chaiyaphum, Thailand; b Department of Information Science, Faculty of Humanities and Social Sciences, Khon Kaen University, Khon Kaen, Thailand

**Keywords:** Trade credit, Trade credit investment, Corporate performance, Agro firms, Thailand, OLS, ordinary least squares, GMM, generalized method of moments, ROA, Return on Assets, SMEs, small and medium-sized enterprises, ROE, Return on Equity, FSIZE, Firm size, LEV, Financial leverage, LQD, liquidity, SGR, sales growth, GDP, economic growth, TC, Trade credit, TC2, The square of trade credit

## Abstract

This paper aims to examine the relationship between trade credit and corporate performance. Empirical evidence on the impact of trade credit investment on firm performance remains unclear. For agro firms, the implications of this relationship have not been thoroughly discussed. Using a panel sample consisting of publicly listed agro firms in Thailand for 2001–2020. The sample set consists of 51 Thai-listed firms with 708 firm-year observations. We employ panel ordinary least squares (OLS) regressions and GMM regressions to obtain the estimation results. We find that going to invest in trade credit increases operating performance significantly, which is what the commercial, financing, and transaction theories of trade credit predicted would happen. Furthermore, cost-benefit analysis should serve as a guide for firms’ trade credit investment decisions. In particular, firms should be aware of the extra cost of trade credit investment and weigh it against the benefits of improved performance.

## 1 Introduction

Trade credit is an important short-term external financing for firms in all sectors, essential to firm life. A loan from a lender to a borrower allows the latter to delay payment when purchasing goods and services and helps the latter reduce short-term financial strain. Trade credit is a useful financial tool for growing firms when favorable terms are agreed upon with a firm's supplier. It is widely practiced as a financing tool to secure the borrower's additional working capital when financial institute credit is inaccessible or limited [1]. Trade credit is a form of short-term borrowing as a result of regular business transactions between companies [2]. Most companies rely on trade credit by borrowing from their providers and lending to their clients [3]; trade credit is not only a widespread regulation but also a spontaneous source and the most source of short-term external finance for firms. Trade credit is less formal than a loan from a commercial bank. Based on a survey database covering 48 countries, on average, 19.7% of all investment financed by external finance sources was done using trade credit [4]. In addition, trade credit is also an instrument to raise sales income, and high risk can lead to higher profitability and increased competitive advantage.

Because trade credit plays a significant role in a firm's operation as a channel of credit, prior studies are related to the relationship between trade credit and firm performance or firm profitability [[5], [6], [7], [8], [9]]. One of the crucial questions in the prior literature is whether a firm's trade credit enhances or weakens its performance. Empirical results in the literature are as inconsistent as the different methodological stances; prior studies presented that the effect of trade credit on profitability is positive. On the other hand, high investment in trade credit is related to high financial costs and risk and thus reduces firm profitability. It is also revealed that this relationship could be positive or negative. The relationship between trade credit and firm performance ratio remains unclear. To fill this gap discussed above, this paper investigates the relationship between trade credit and firm performance in the agro-industry. The agro-industry is of particular interest because agro-products have inelastic demand; they are fragile products characterized by quickly perishable, difficult to control both quality and quantity of output but can readily be verified for quality. It is argued that the product may likely have unpredictable changes in demand or production cost; suppliers of fast-changing products are more likely to require cash. Compared to trade credit sales, cash sales will identify trade transactions in the agro-industry [10]. So, the question arises, whether trade credit investment is related to the profitability of agro firms, what does that relationship look like? This question has been mostly uncleared in the empirical trade credit literature as industry-level studies are rare, particularly for the agro-industry; trade credit investment in this industry is assumed to be lower than in non-agro industries like the manufacturing industry [7].

The main objective of a business is to earn its operating performance. Operating performance is based on how well assets are turned into profits and resources are used to make money. Accounting-based performance measure, Return on Assets (ROA), is widely used as a performance indicator calculated from a firm's financial statements [11,12]. Financial statements are a significant part of the information set provided by firms to investors. Investors might contrast ROA with the amount of interest firms pay on their debt. It is unreliable if a firm makes less money from its investments than it spends to finance them. To gauge the realized returns on the firm's growth objectives, investors might compare ROA to the firm's cost of capital. A firm should obtain ROA that surpasses capital costs before embarking on expansions that increase shareholder wealth. ROA shows the earnings generated from capital assets that have been invested.

In this paper, we used to agro-list companies in the Stock Exchange of Thailand as a research sample to investigate the effect of trade credit on corporate performance. Thailand is a significant producer of rice and a major exporter of agricultural goods, but the agro-industry still faces many challenges. We argued that there is a need to learn more about activities that add value in order to make it easier and more efficient for companies in the agro-industry to meet their goals. We chose to examine a sample of publicly listed agro-industry firms in Thailand for three reasons. First, the importance of trade credit as a financing source is even more notable in emerging countries where access to external finance is restricted [13]. Regarding the trade credit situation, 96% of trade in Thailand is through credit and 4% in cash [14]. Second, in Thailand, agriculture is a highly competitive and diverse sector. Agriculture accounts for 6% of Thailand's GDP but employs around one-third of the country's labor force. Since Thai agriculture plays a key role in Thailand's development, agriculture provides many career opportunities for Thai citizens [15]. Finally, Thailand's literature on trade credit is still scare and has received less attention. To the best of our knowledge, only [16] explored the association between trade credit and firm performance of listed SMEs in East Asia and the Pacific, namely, China, Vietnam, Malaysia, Thailand, Japan, South Korea, Taiwan, Singapore, and Hong Kong, and [17] investigated the association between trade credit and profitability of small and medium-sized enterprises (SMEs) from nine countries or territories located in the East Asia and Pacific region. Thus, it is essential to provide further evidence for this issue in Thailand. We discussed the influence of trade credit on firm performance within emerging economies represented by Thailand. Moreover, [18] investigated the role of information sharing in trade credit allocation in Thailand. Therefore, it is essential to provide further insight into this topic in Thailand.

The remainder of the paper is structured as follows. In Section 2, we provide an overview of related theories and propose a hypothesis. Section 3 explains our data, variables, and research methodology. Section 4 presents empirical results. Finally, Section 5 concludes the paper and provides suggestions for further research.

## 2 Literature review and hypothesis development

In the last few decades, there has been more and more research on how trade credit affects business improvement. Trade credit is defined as the ratio of trade receivable divided by total assets. It is a credit between trading partners who buy and sell products and services. The seller agrees to let the buyer get the product or service first and pay for it later, based on trade credit terms. This helps the buyer buy more because it gives them more money to spend. Trade credit also includes times when the buyer pays the seller ahead of time for goods and services. Generally, the term of payment agreed upon between the buyer and the seller for trade credit and advance payment is short-term (i.e., no more than one year). A supplier agreeing to bill a client could lead to new partnerships and bigger contracts.

Nevertheless, trade credit has three main differences from other loans for business operators. First, suppliers’ credit is not in the form of cash. Second, trade credit is issued by non-financial firms. Finally, trade credit between buyer and seller does not enter into a rigorous formal contract while lending or issuing debt instruments by financial institutions and the private sector, which requires a strict and formal contract between the lender and the borrower [19]. Trade credit providers have a credit policy outlining their terms. Understanding the contract conditions is essential, whether giving or getting trade credit. Customers with a cash flow should request early payment discounts from many businesses that provide them. A typical discount is 3/10, n/45, which means that you can get a 3% discount if you pay within ten days of getting the invoice, and the rest of the balance must be paid by the end of 45 days after that.

The trade credit theories explain the connection between trade credit and how well a business operates. These theories are the financing, commercial, and transaction cost theories. In the commercial aspect, prior studies have shown that suppliers will offer trade credit to clients to increase sales volume and profits when the cost of raising funds from capital markets is extremely high. Trade credit provides insight as a strategic investment in attracting and retaining client loyalty, building a more stable client base, and generating consistent future demand and revenue for the firm's products and services [20]. Reference [21] suggested that suppliers lend to constrained businesses because they have a comparative advantage in learning about potential customers and can more efficiently liquidate assets. Businesses with easier access to credit extend more trade credit, thus generating additional revenues and receiving more profits. Furthermore, a firm's profitability and competitiveness can be improved by trade credit supply through increasing market share [22,23]. By giving trade credits to current trading partners, a company can keep selling products and maintaining supply chains as long as the partner can still pay the debt on time. The seller gives the customer a good product by the agreed-upon date, and the customer pays for the product by the agreed-upon date. To get customer retention, suppliers must develop a reputation for high-quality goods and take advantage of trade credit. Therefore, both partners have incentives to continue partnering. From a business point of view, trade credit investments can be used as a way for companies to build their brands [8].

Reference [24] developed the financial motivations for using trade credit and contended that financially sound businesses would increasingly extend trade credit to maintain relationships with smaller clients during a credit crunch. The supplier operates as a financial middleman for clients with restricted access to capital markets, thereby funding their clients’ expansion. When raising capital from the financial markets is expensive due to restrictive monetary policy, businesses may offer trade credit to clients to boost sales and profits. Financial market imperfections allow sellers to outperform financial intermediaries in terms of information and cost of collection. Firms can boost their earnings by investing in trade credit to produce additional returns. Firms with extra money could invest in trade credit to get interest income, comparable with they could invest in securities that can be sold to get better returns and make more money [25]. Extending trade credit could strengthen the relationship with its suppliers, especially when consumers face temporary shocks to their cash flow that could threaten their ability to stay in business. Because of this, firms with more cash will give trade credit to firms with less cash instead of investing, easing the financial pressure on clients.

The boundaries of credit policy are set by rising opportunity costs and flaws in the financial system. Assuming that the marginal revenue from trade credit lending equals the marginal cost, it leads to the optimal amount of accounts receivable. Nevertheless, giving out additional trade credit exposes a firm to the financial risks of client non-payment, delayed payment, and default.

In previous studies in the context of transaction cost theory, businesses can boost their profits by reducing the cost of conducting economic transactions by employing trade credit [25]. By offering trade credit to stimulate demand during times of low demand and increase the volume of transactions, firms can also increase transactional efficiency. Trade credit supply may decrease the storage expenses involved with maintaining extensive inventories and the volume of bank transactions and related costs [24,25]. Therefore, trade credit could encourage sales by easing trade credit terms during times of low demand, enabling it to smooth out irregular demand.

Even though there are benefits to trade credit, costs are also involved. Administrative costs come with giving trade credit, such as costs for regulation, monitoring, and managing accounts receivable [10,22]. Firms that invest in trade credit are subject to financial risks like missed or delayed payments. The opportunity cost of credit sales, as opposed to cash transactions, also exists. In other words, funds used to finance client purchases could be invested in different short-term ventures.

Consequently, if the cost of engaging in trade credit outweighs the benefits, firm profitability may decrease as a function of trade credit investment [20]. A massive trade credit provision holds up much of the capital in trade receivables. This could be challenging for companies to invest in projects that add value because they do not have enough money. In this case, the company might have to go to the capital market to get more money at a higher cost. It could be claimed that investing in trade receivables has costs that outweigh the benefits; as a result, a firm that maintains a high level of receivables will have smaller profits. When the costs and advantages of investing in trade credit are equal, trade credit investment is at its finest [22,25]. Because there are many accounts receivable in the sector, it has become harder to deal with all the risks that come with credit policies. There might come a time when there is a negative relationship between trade credit investment and profitability. Investments in trade credit initially enhance businesses’ earnings. However, since working capital would be involved in the trade credit investment, the equity cost would rise. Also, the amount of trade credit firms invest is directly related to the amount of money they spend on administration.

The relationship between trade credit investment and profitability may be negative if the costs of engaging in trade credit outweigh the advantages, including the possibility of bad debts. Nevertheless, trade credit theory will support a positive link between trade credit investment and profitability. This basic hypothesis is based on the idea that firms weigh the costs and benefits of trade credit when deciding how much to invest in maximizing profits. Trade credit may challenge the possibility of growth for the firms. Based on the above discussion, we propose that our main hypothesis be expressed as follows:

Hypothesis: Trade credit is positively associated with firm performance.

## 3 Research methodology

### 3.1 Data

This study looked at the finances of Thai agribusiness companies listed on the Stock Exchange of Thailand (SET) from 2001 to 2020. The data comes from the SETSMART database and the Securities and Exchange Commission's website. After taking out the missing key data, the final unbalanced panel sample set has 708 firm-year observations for 51 agro firms. All variables are winsorized at the 5% and 95% levels to minimize the effect of outliers. The descriptive statistics of the data are displayed below.

### 3.2 Variables description

#### 3.2.1 Dependent variable

A description of all variables is presented in table form in Appendix 1 section. Consistent with the finance and business literature, we used return on assets (ROA) as a proxy to evaluate firm performance, the dependent variable. Following previous explorations, ROA is the finest indicator of corporate performance or corporate profitability [8,26,27]. ROA is measured as the ratio of earnings before interest and taxes (EBIT) to total assets. As a robustness test, we also used Return on Equity (ROE), calculated as the ratio of earnings before interest and taxes to equity, as an alternative way to measure a company's performance. ROE is influenced by the number of shares outstanding, with 83.80% of firms clearly adopting ROE as a performance measure [28] ROE is a measure of a firm's profitability and how efficient it is in originating profits. The higher the value of a firm's ROE, the more probably investors will invest in it. Consequently, ROE is a measure that shows an investor how much profit can be generated by the firm using the capital invested by its shareholders [29].

Trade credit and the square of trade credit serve as the main explanatory variables. According to the research of [7,27,30], trade credit (TC) is expressed as the ratio of trade receivable to total assets. It is a ratio that evaluates the proportion of a firm's assets maintained as trade credit receivables. The trade credit square (TC2) is included to test for a non-linear relationship between trade credit investment and corporate performance. TC2 is mean-centered to reduce collinearity [7].

Furthermore, the firm's profitability can be impacted by various firm-specific factors. These control variables are taken into account in this investigation. The following control variables are included in regression analyses: Firm size (FSIZE) is computed as the natural logarithm of total assets [7,31,32]. Existing research has yielded a variety of findings regarding the relationship between firm size and firm profitability. Financial leverage (LEV) is measured as the ratio of total liabilities to total assets. The natural logarithm of cash and short-term investment ratio to all assets is used to measure liquidity (LQD). It is anticipated that businesses with increased liquidity will make more money since they can take advantage of beneficial possibilities without borrowing money from the financial markets. As specified by the previous literature, sales growth (SGR) is computed as sales growth, and economic growth (GDP) is computed as the yearly gross domestic income growth rate. These variables capture a wide range of firm characteristics that may be correlated with firms' performance and use of trade credit. Please see all variables and their definitions in detail in Appendix 1.

### 3.3 Empirical methodology

#### 3.3.1 Panel OLS estimations of corporate performance

In this section, we explain how the model was built to test the positive relationship between trade credit and the performance of agro firms. We provide a technique for data analysis that might be used to answer our research question and verify our hypothesis. Consider that the main objective of our research is the impact of trade credit on firm performance. We examined the firm as our unit of analysis to better understand how variations in trade credit affect firm performance. Our initial method of estimation is ordinary least squares (OLS). We estimated a panel OLS regression of trade credit on firm performance and a set of control variables to assess the effect of trade credit on firm performance. We integrated firm fixed effects to account for omitted time-invariant firm characteristics and time fixed effects to account for any unrecognized time-variant effect that impacts each firm in the sample, as necessary. We then used the results of the fixed effects estimation to assess the effect of trade credit on the performance of agricultural firms.

We used a fixed effects regression to estimate the effect of trade credit on firm performance. The fixed effects model includes a set of dummy variables that indicate the time the observation was made for each firm. The model also includes firm fixed effects, estimated as a set of dummy variables that indicate the firm to which the observation belongs. The fixed effects model controls for omitted time-invariant firm characteristics and any unrecognized time-variant effect that impacts each firm in the sample. To generate the empirical results based on panel data for our hypothesis, we employed the panel OLS estimations, often used in the finance literature [7,8]. The following equation model as expressed in Eq. (1).

(1) *Y*_*i,t*_ = *β*_*0 +*_*β*_*1*_*TC*_*i,t*_ + *β*_*2*_*TC2*_*i,t*_ + *β*_*3*_*FSIZE*_*i,t*_ + *β*_*4*_*LEV*_*i,t*_ + *β*_*5*_*LQD*_*i,t*_ + *β*_*6*_*SGR*_*i,t*_ + *β*_*7*_*GDP*_*i,t*_ + *μ*_*i*_ + *ɸ*_*i*_ + *ϵ*_*i,t*_

where *Y*_*i,t*_ is ROA, performance of firm i at time t, *β*_*0*_ is the intercept term, and *β*_*1*_*–β*_*7*_ are parameters relating to the explanatory/control variables to be estimated. *TC2*_*i,t*_ is added to test for a non-linear relationship between trade credit and firm performance, and the other variables are controls. All other variables are as defined previously. *μ*_*i*_ is the dummy variable for the period 2001–2020, *ɸ*_*i*_ is the dummy variable for sub-industry group, and *ϵ*_*i,t*_ is the independent and identically distributed error term.

#### 3.3.2 GMM estimations of corporate performance

In our study, the main issue with empirical evidence is the possibility that the measure of trade credit and the unobservable random disturbance term in regression will be correlated (trade credit is endogenous in an equation). Assume that a firm with higher performance prefers to invest in more trade credit to boost profits because it can do so with its available funds compared to a firm with poor performance. Because trade credit is a function of itself, this could be a case of simultaneity bias or backward causation. In a model with continuous variables, an endogeneity issue arises when there is significant simultaneity bias or reverse causality. Reference [33] found that the generalized method of moments (GMM) method could always get accurate results for panel data sets while considering different types of endogeneity. A first-difference GMM was initially proposed by Ref. [34] to address this problem and has been widely applied in many empirical studies. GMM estimation and/or lagging the explanatory variables have traditionally been used to address this problem [31]. We used GMM estimation to generate empirical results for our hypothesis while mitigating the endogeneity problem. We select the first lagged variables, TCt-1 and TC2t-1, as our instrumental variables following [7,8] because exogenous variables are insufficient to ensure firm performance.

## 4 Empirical results

### 4.1 Summary statistics

Respectively, we demonstrate the descriptive and correlation matrix in Tables 1 and 2. Table 1 shows a summary of key statistics for the final sample of 708 firm-year observations from 51 firms used in this study. For the period 2001–2020, the average ROA ratio is around 7% and that the average value of ROE ratio is about 12%. These statistics indicate that the average rate of return for Thai firms is probably lower than most people would have predicted. Almost all of the sample firms participate in trade credit. In our sample, 96.08% of firms have trade credit accounts, indicating that this is a typical short-term financing strategy in the Thai public agro-industry. On average, firms invest 12% of their total assets in trading credit. Suggesting that the public agro industry in Thailand uses trade credit as a common method of short-term financing. Comparatively, this is in line with the 12% [7] reported for the US agro-food industry. The mean value of financial leverage and sales growth is 55% and 10%, respectively. In view of the data provided, it appears that a firm invests an average of 12% of its total assets in trade credit. This is lower than the 29% reported by Ref. [35] for the Spanish agro-food industry and lower than the typical range for European SMEs and Vietnamese enterprises [8]. European nations tend to place highly in trade credit investment, as European SMEs and Vietnamese enterprises are able to maximize the use of trade credit in order to increase their profitability due to their scale and expertise relative to Thai firms. In contrast, the majority of Thai agro firms appear to be unqualified to manage bargaining power to extend their payment periods and trade credit strategy. Reference [36] recommend that firms should limit the ratio of trade credit to total assets to 19% in order to expand as quickly as possible.

**Table 1:** Summary statistics for the sample.

Variable	Mean	Minimum	Maximum	S.D.	Observations
ROA	0.07	−1.43	0.43	0.12	708
ROE	0.12	−3.81	0.85	0.27	708
TC	0.12	0.00	0.91	0.09	708
TC2	0.02	0.00	0.82	0.05	708
FSIZE	15.35	11.21	20.45	1.36	708
LEV	0.55	0.00	29.13	1.90	708
LQD	−2.25	−14.15	0.00	3.35	708
SGR	0.10	−0.98	10.63	0.56	708
GDP	0.03	−0.06	0.31	0.04	708

**Table 2:** Correlation matrix between variables.

	1	2	3	4	5	6	7	8	9
**1. ROA**	1								
**2. ROE**	0.677***	1							
**3. TC**	−0.096***	−0.005***	1						
**4. TC2**	−0.189***	−0.079***	0.869***	1					
**5. FSIZE**	0.178***	0.154***	−0.213***	−0.230***	1				
**6. LEV**	−0.282***	−0.029***	0.008***	0.001***	−0.118***	1			
**7. LQD**	0.024***	0.047***	−0.025***	−0.041***	−0.047***	0.044***	1		
**8. SGR**	0.119***	0.089***	0.048***	0.019***	−0.010***	0.048***	−0.015***	1	
**9. GDP**	0.080***	0.065***	0.126***	0.106***	−0.152***	0.058***	−0.092**	0.167	1

Note: Symbols *, **, and *** represent statistical significance at the 10%, 5%, and 1% levels, respectively.

The mean value for financial leverage (LEV) is 55%, indicating that the Thai Firms in our sample have lower degrees of financial leverage than US firms (the mean value of leverage = 60.9%) as reported by Ref. [7].

### 4.2 Correlation matrix

Table 2 presents the Pearson correlation matrix of all variables. The Pearson correlation coefficients are low. Since most correlations are less than 0.20, the problem of multicollinearity is not a great concern. The sign of the correlation coefficient in the given case is generally in line with expectations. As expected, ROA and ROE are highly correlated (r = 0.68), which proves that they can be used almost interchangeably as a proxy for firm performance.

### 4.3 Results of panel OLS regressions

In this section, we show our empirical results about how trade credit affects a firm's performance. Table 3 shows the results of a panel OLS regression with firm performance (ROA) as the dependent variable. The ratio of earnings before interest and taxes (EBIT) to total assets was used to figure out how well a company operates. Column (1) reveals the results of our baseline regression. We find that firm size (FSIZE) and sales growth (SGR) are positively associated with firm performance. These findings are consistent with [37], which revealed a positive relationship between firm size, sales growth, and firm performance. The coefficient on firm size (FSIZE) is positive and statistically significant, suggesting that larger firms tend to have better operating performance. Additionally, the coefficient on sales growth (SGR) is also positive and statistically significant at the 1% level, indicating that agro firms that have higher sales growth tend to have better operating performance. However, trade credit (TC) negatively affects firm performance. The coefficient on trade credit is −0.095, which is statistically different from zero at the 5% level. This means that if the amount of trade credit goes up, the performance of the firm will go down. This result backs up a hypothesis that there is a positive relationship between trade credit and a company's performance. The results of earlier studies (e.g. Refs. [[6], [7], [8], [9]]) found a positive correlation between trade credit and firm performance, which is in contrast to this one. The coefficient on leverage (LEV) is negative and statistically significant at the 1% level, suggesting that agro firms that have higher leverage tend to have poorer operating performance. This means that higher leverage (more debt) results in a decrease in operating performance. On the other hand, liquidity (LQD) and gross domestic income growth rate (GDP) are not associated with operating performance, it means that there is no relationship between liquidity, GDP and operating performance.

**Table 3:** Panel OLS regressions of firm performance.

Variable	(1) ROA	P-value	(2) ROA	P-value
Constant	−0.095 (0.050)	0.057	−0.099** (0.049)	0.043
TC	−0.095** (0.046)	0.041	0.343*** (0.090)	0.000
TC2			−0.993*** (0.174)	0.000
FSIZE	0.012*** (0.003)	0.000	0.010*** (0.003)	0.001
LEV	−0.017*** (0.002)	0.000	−0.017*** (0.002)	0.000
LQD	0.002 (0.001)	0.195	0.001 (0.001)	0.285
SGR	0.027*** (0.007)	0.000	0.026*** (0.007)	0.000
GDP	0.113 (0.113)	0.318	0.103 (0.111)	0.353
*R*^*2*^	0.127		0.165	
Adjusted *R*^*2*^	0.119		0.157	
*F*-statistic	16.978***		19.827***	
Observations	708		708	

Note: This table presents the results of panel OLS regression of firm performance. The dependent variable is the return on assets (ROA), measured as the ratio of earnings before interest and taxes to total assets, for a sample of 51 firms during the period 2001–2020. Please see Appendix 1 for the variable description. Robust standard errors, which are clustered at the firm level, are reported in parentheses. *, **, and *** represent statistical significance at the 10%, 5%, and 1% levels, respectively.

The quadratic term of trade credit (TC2) is presented in column (2). In the panel OLS estimate, TC is still significant but is linked to firm performance in a positive association. Additionally, TC2 is significant and inversely associated with firm performance. The TC coefficient is 0.343, which is statistically different from zero at the 1% level. This means that a firm's operating performance will improve if it has more trade credit. Moreover, the firm size (FSIZE) and the sales growth (SGR) coefficients all continue to be positive and statistically significant, but the leverage (LEV) coefficients are negative and statistically significant. However, the coefficient on LQD and GDP are positive but insignificant, indicating that liquidity and gross domestic income growth rate are not associated with firm performance, which means that there is no relationship between liquidity, GDP and firm performance.

### 4.4 Results of GMM regressions

As explained in Section 3.3.2 of the study, the results of panel OLS regression can be biased by the endogeneity issue that commonly affects the dependent variables. To mitigate this concern and detect the causal effect of trade credit on firm performance, the study adopted GMM estimation and also used it to test the robustness of the findings.

Table 4 presents the GMM results using ROA as the dependent variable. In order to determine whether the validity of our instrument set can be rejected, we use the Sargan test (for over-identifying restrictions). The instrumental variable approach is the most efficient method to account for all types of endogeneity if suitable instruments can be found [2]. The tests' results indicate that none of the models in Table 4, Table 6 can have the over-identifying restrictions rejected at the 5% level, indicating that the instruments used in this study are suitable. Column (1) of Table 4 presents the baseline model that includes only TC while column (2) includes TC2. We find that the coefficient on trade credit (TC) is negative and statistically significant at the 1% level, implying that agricultural firms with lower trade credit tend to have better operating performance. Larger firms appear to have higher operating performance because the coefficient on firm size (FSIZE) is statistically positive. The coefficient on sales growth (SGR) is positive and statistically significant at the 1% level, suggesting that agro firms with higher sales growth tend to have better operating performance. The coefficient on leverage (LEV) is negative and statistically significant at the 1% level, implying that agricultural firms with higher leverage tend to have lower operating performance. Nevertheless, the coefficients on LQD and GDP are positive but insignificant, showing that liquidity and the gross domestic income growth rate are not associated with firm performance. The quadratic term of trade credit (TC2) in column (2) of their analysis. Using GMM regressions, the study found that TC is still significant and positively associated with firm performance after being instrumented, consistent with previous estimations. Additionally, the study found that TC2 is significant and inversely associated with firm performance. The coefficient for TC is 0.482, which is statistically different from zero at the 1% level. This implies that increasing a firm's trade credit will improve its operational performance, an increase in trade credit by one unit results in a 0.482 increase in firm performance. This finding is consistent with the research of [38] which found that firm value and trade credit have a positive correlation at low levels of receivables and a negative correlation at high levels among Spanish listed companies. Prior studies such as [17] have suggested that companies that can increase their investment in trade credit can boost their profitability, and the effect is more pronounced for financially unrestricted firms (more liquid, larger firms), firms facing inflationary pressures, and firms with a larger market share. Furthermore, these studies also suggest that there are variations in the profitability of trade credit depending on operational, financial, and commercial purposes. However, other studies such as [39] have found that the level of trade credit is not correlated with firm performance. They found that when they used the instrumental variable approach to account for endogeneity, the statistical insignificance, casting doubt on the claim that trade credit improves firm performance. This could be due to the fact that trade credit may have a long-term impact on firm performance rather than a short-term impact. Studies such as [40] have found a negative relationship between trade credit and firm performance, which implies that companies may benefit from external debt or equity financing at a lower cost than trade financing. While [8] found a nonlinear relationship between trade credit investment and the profitability of Vietnamese manufacturing companies. They suggest that businesses can enhance performance and boost profits by utilizing reasonable levels of trade credit, and if they refine their trade credit investment strategy, they can increase their profits.

**Table 4:** GMM regressions of firm performance.

Variable	(1) ROA	P-value	(2) ROA	P-value
TC	−0.169*** (0.055)	0.002	0.482*** (0.145)	0.000
TC2			−1.529*** (0.346)	0.000
FSIZE	0.006*** (0.000)	0.000	0.003*** (0.001)	0.000
LEV	−0.017*** (0.002)	0.000	−0.018*** (0.002)	0.000
LQD	0.002 (0.001)	0.206	0.001 (0.001)	0.386
SGR	0.028*** (0.007)	0.000	0.025*** (0.007)	0.000
GDP	−0.092 (0.112)	0.414	0.083 (0.111)	0.455
Sargan statistic	2.116		4.909	
Sargan *p*-value	0.146		0.086	

**Note:** This table presents the results of GMM regression of firm performance. The dependent variable is the return on assets (ROA), measured as the ratio of earnings before interest and taxes to total assets, for a sample of 51 firms during the period 2001–2020. Please see Appendix 1 for the variable description. Robust standard errors, which are clustered at the firm level, are reported in parentheses. *, **, and *** represent statistical significance at the 10%, 5%, and 1% levels, respectively.

The firm size (FSIZE) and the sales growth (SGR) coefficients are both positive and statistically significant, which suggests that larger firms have more advantages than smaller firms in improving their operating performance. They also found that the leverage (LEV) coefficients are negative and statistically significant. This finding is consistent with the results from other investigations such as [17,41]. Additionally, the study found that the coefficient on LQD is positive and GDP is negative but insignificant.

### 4.5 Robustness test

This section explores the results of some extra tests to see how well the main findings hold up. ROA, an accounting-based measure of how well a company operates, is used to figure out how profitable a company is. Using ROE, a performance measure based on accounting, the relationship between trade credit and firm performance is tested again to see if it is strong. Table 5 presents a panel OLS regression of ROE for the full sample. As seen in Table 5, column (1), the results show that the coefficient signs for trade credit are positive but insignificant. The coefficients for the size of the firm (FSIZE), the growth of sales (SGR), and the growth of the economy (GDP) are all positive and statistically significant. The pattern of results is generally similar, which is consistent with our expectations. Trade credit's quadratic term (TC2) is displayed in column (2). In the panel OLS calculation, the TC is still statistically significant and linked to firm performance in a positive way. Furthermore, TC2 is also significant and negatively associated with firm performance. The sign of variables FSIZE and SGR are also similar to those in estimations of previous sections.

**Table 5:** Panel OLS regressions of trade credit on ROE.

Variable	(1) ROE	P-value	(2) ROE	P-value
Constant	−0.414*** (0.121)	0.000	−0.420*** (0.120)	0.001
TC	0.058 (0.112)	0.608	0.723*** (0.219)	0.001
TC2			−1.505*** (0.428)	0.001
FSIZE	0.034*** (0.008)	0.000	0.031*** (0.007)	0.000
LEV	−0.003 (0.005)	0.587	−0.003 (0.005)	0.539
LQD	0.005 (0.003)	0.079	0.005 (0.003)	0.107
SGR	0.037** (0.018)	0.038	0.035 (0.018)	0.053
GDP	0.594** (0.273)	0.030	0.579** (0.271)	0.033
*R*^*2*^	0.042		0.059	
Adjusted *R*^*2*^	0.034		0.050	
*F*-statistic	5.173***		6.276***	
Observations	708		708	

Note: This table presents the results of GMM regression of firm performance. The dependent variable is the return on equity (ROE), measured as the ratio of earnings before interest and taxes to equity, for a sample of 51 firms during the period 2001–2020. Please see Appendix 1 for the variable description. Robust standard errors, which are clustered at the firm level, are reported in parentheses. *, **, and *** represent statistical significance at the 10%, 5%, and 1% levels, respectively.

The results from Table 6 of the GMM regressions, using ROE as the dependent variable, are generally similar to those presented in Table 5. Column (1) of Table 6 presents the baseline model that includes only TC while column (2) includes TC2. The study finds that the coefficient on trade credit (TC) is negative and statistically significant at the 1% level. The coefficient on firm size (FSIZE) and sales growth (SGR) is positive and statistically significant at the 1% level, indicating that agro firms with higher total assets and sales growth typically perform better operating performance. Furthermore, the coefficient on LQD is positive and GDP is negative but insignificant.

**Table 6:** GMM regressions of trade credit on ROE.

Variable	(1) ROE	P-value	(2) ROE	P-value
Constant	−0.414*** (0.121)	0.000	−0.420*** (0.120)	0.001
TC	0.058 (0.112)	0.608	0.723*** (0.219)	0.001
TC2			−1.505*** (0.428)	0.001
FSIZE	0.034*** (0.008)	0.000	0.031*** (0.007)	0.000
LEV	−0.003 (0.005)	0.587	−0.003 (0.005)	0.539
LQD	0.005 (0.003)	0.079	0.005 (0.003)	0.107
SGR	0.037** (0.018)	0.038	0.035 (0.018)	0.053
GDP	0.594** (0.273)	0.030	0.579** (0.271)	0.033
*R*^*2*^	0.042		0.059	
Adjusted *R*^*2*^	0.034		0.050	
*F*-statistic	5.173***		6.276***	
Observations	708		708	

Note: This table presents the results of GMM regression of firm performance. The dependent variable is the return on equity (ROE), measured as the ratio of earnings before interest and taxes to equity, for a sample of 51 firms during the period 2001–2020. Please see Appendix 1 for the variable description. Robust standard errors, which are clustered at the firm level, are reported in parentheses. *, **, and *** represent statistical significance at the 10%, 5%, and 1% levels, respectively.

Column (2), TC is still significant but is linked to firm performance in a positive association. Additionally, TC2 is significant and inversely associated with firm performance. Moreover, the firm size (FSIZE) and the sales growth (SGR) coefficients all continue to be positive and statistically significant, but the leverage (LEV) coefficients are negative and statistically significant. Furthermore, the coefficient on LQD is positive and GDP is negative but insignificant.

## 5 Conclusion

This study has examined the relationship between trade credit and firm performance. Trade credit management is an essential part of corporate strategy and working capital policy. Our sample set has firm-year observations for agro companies listed on the Stock Exchange of Thailand from 2001 to 2020. The above empirical findings not only provide a quantitative description of the relationship between trade credit investments and the performance of firms, but they also give us a reason why certain economic things happen in the Thai agro-industry The majority of the firms in the sample invest in trade credit. However, the level of trade credit investment in Thailand's public agro industry varies. In order to examine the relationship between trade credit investment and the operating performance of firms, the study used econometric estimation methods such as panel OLS and GMM estimation. The estimates imply that trade credit investment significantly impacts firm performance. In this study, we find that the result match up with the initial findings, but [16] claimed that a company's profitability decreases when its trade credit level is not at the right level.

In addition, this study is the first to report that trade credit has a positive effect on firm performance of agro-industry in Thailand. Food is absolutely essential to human existence. Given that food is necessary for human survival, the agricultural sector is the only one whose product demand could never be insufficient. Based on what we have find, companies with a lot of trade credit tend to have a higher ROA ratio. This indicates that high trade credit holdings or high trade credit efficiency may be an indicator of a firm's decreasing performance. Firms should attempt to maintain trade credit investment levels as close as feasible to the ideal point to prevent their profitability from declining. Due to the effects of several variables that change over time, such as the opportunity cost of capital, the rate of customer default, or the effect of toxic loans on their trade credit, it is usually hard for firms to figure out their ideal trade credit level [22]. Credit expansion increases the number of unpaid accounts receivable; hence, firms must monitor these clients to ensure they make their payments on time. A firm that conducts its business in cash does not face this problem. Firms that extend trade credit face the risk of not getting paid back on time according to the terms of their clients. Then, if they are unlucky, they have to chase the payments, which hurts their cash flow. Suppliers must keep an eye on potential losses, and clients should not overestimate their ability to pay.

This study has both academic and practical implications. This is the first academic study to examine how trade credit affects how well Thai agro-businesses do. The conclusion of our study could help managers, business owners, and investors understand how a company's current credit policy affects its success. Firms need to consider financing, commercial, and transaction cost theories to improve their trade credit investments. Using only one theory may not be enough to account for the different ways different firms are set up. In the same way, regulators and policymakers can use this study as a guide to develop better internal trade credit strategies. These strategies balance risk and profit and are a key part of how a company manages its finances. The greatest amount of credit that can be extended at this time should be taken into account by policymakers since, beyond that, the effect will change. The study still contains limitation, we use information gathered from the Stock Exchange of Thailand. Companies in our sample are listed agro firms, so the study still has limitations. This means that a sizable number of other listed companies remain out of our research, and subsequent works on the subject should focus on more specialized industries. This can assist businesses in creating specific credit-granting strategies.

Lastly, future research could take into account including the cost of capital measure to test whether the association between trade credit and firm performance may be influenced by the cost of capital since a firm's trade credit investment decisions and outcomes may feasibly be powered by the cost of capital.

## Author contribution statement

Wirapong Chansanam: Analyzed and interpreted the data; Contributed reagents, materials, analysis tools or data; Wrote the paper.

Umawadee Detthamrong: Conceived and designed the experiments; Performed the experiments; Analyzed and interpreted the data; Wrote the paper.

## Funding statement

This research did not receive any specific grant from funding agencies in the public, commercial, or not-for-profit sectors.

## Data availability statement

None.

## Declaration of interest's statement

The authors declare no conflict of interest.

## Figures and Tables

**Table:** 

Variable name	Variable code	Description
Trade credit	TC	The ratio of trade receivable to total assets.
The square of trade credit	TC2	The square of trade credit.
Return on Assets	ROA	The ratio of earnings before interest and taxes (EBIT) to total assets.
Return on Equity	ROE	The ratio of earnings before interest and taxes to equity.
Firm size	FSIZE	The natural logarithm of total assets.
Financial Leverage	LEV	The ratio of total liabilities to total assets.
Liquidity	LQD	The natural logarithm of the ratio of cash and short-term investment to total assets.
Sales growth	SGR	The ratio of (Sales at *t*-sales at *t* − 1)/sales at *t* − 1.
Economic growth	GDP	Yearly gross domestic income growth rate.

## Appendix 1 Variable description

This table presents definitions of key variables.
